# AAC and Autism: Manual Signs and Pecs, a Comparison

**DOI:** 10.3390/bs12100359

**Published:** 2022-09-26

**Authors:** Alessandro Frolli, Sonia Ciotola, Clara Esposito, Sara Fraschetti, Maria Carla Ricci, Francesco Cerciello, Maria Grazia Russo

**Affiliations:** 1 Disability Research Centre, University of International Studies of Rome, 00147 Rome, Italy; 2 FINDS—Italian Neuroscience and Developmental Disorders Foundation, 81040 Caserta, Italy

**Keywords:** ASD, PECS, MS, augmentative alternative communication, language

## Abstract

Autism Spectrum Disorders (ASD) represent a heterogeneous group of disorders, with onset in developmental age, which present a clinical expressiveness that varies from subject to subject and in the same subject over time. The DSM 5 defines Autism Spectrum Disorders according to two main criteria: persistent deficits in social communication and social interaction in multiple contexts and limited and repetitive patterns of behavior, interests or activities. This disorder can manifest itself across a broad spectrum of severity levels. Indeed, ASD includes clinical conditions from low functioning (LF—Low Functioning) to high functioning (HF—High Functioning), taking cognitive and adaptive functioning as a reference. One of the main characteristics of individuals with ASD is a delay in receptive and expressive communication. These deficits have led to the identification of evidence-based practices, particularly for those with severe communication difficulties. Augmentative Alternative Communication (AAC) has been implemented to compensate for deficits in functional communication and language skills in individuals with complex communication deficits. The AAC comprises communication systems including the Manual Signs, speech and image output devices (Communicators), and Image Exchange Systems (PECS); these systems have been shown to actually improve various abilities in autism such as social skills, modify and improve dysfunctional behaviors and, above all, improve learning. Recent meta-analyses have shown how PECS and Manual Sign can have great effects on the communication skills of young people with autism. The aim of this study is to compare these two types of intervention to improve communication in terms of vocalization in subjects with ASD and try to understand which of the two lead to more significant and rapid improvements.

## 1. Introduction

Autism Spectrum Disorder (ASD) is one of the most complex developmental disabilities, due to the marked impairment of communication skills [1,2]. Studies estimate that only 30% of people with autism are minimally verbal. It could be crucial to identify factors that predict longer-term expressive development in children with language delays in order to identify treatment for those who are at risk of language impairments. After the 1990s, several studies claimed that only 10–15% of children with autism are minimally verbal. It is unclear whether the previous data were higher due to inaccurate diagnoses or the lack of specific treatments [3]. This change is due in part to widening diagnostic criteria, from early diagnosis and greater access to more effective early interventions that significantly improve spoken language and communication skills in preschool children with ASD, thereby preventing them becoming non-verbal at a later age [4].

ASD is characterized by social communication deficits and limited and repetitive behaviors [1,2]. The ability to communicate effectively is an essential life skill, and it can have several negative outcomes, including poor academic performance, behavioral difficulties, and reduced quality of life. Over the past few decades, a large body of research has shown the effectiveness of AAC for people with ASD. Due to the inability of many people with autism to acquire speech, researchers studied a variety of augmentative and alternative communication systems. To improve the limitations of existing augmentative systems, such as hand signs and image systems, the Picture Exchange Communication System (PECS) was developed. PECS is a communication method based on the exchange of iconic images for the purpose of requesting, tagging and commenting. PECS is different from other communication systems [5] since images are easier to understand and more comprehensible. They do not require any specific previous ability to process them. Manual signs and voice training programs commonly require participation (i.e., eye contact) and imitation, both of which are skills that could be difficult to acquire [6]. PECS consists of the delivery of iconic images to communicate, which helps in avoiding participation, imitation, pointing or matching skills. In this study, the two main systems of AAC used in autism were considered, namely the PECS system and manual signs. Specifically, we considered 82 children with ASD level 1 who had not developed language yet; the sample was divided into two subgroups that underwent specific AAC training. The results of the PECS group were analyzed and compared with those of the manual signs training Group. The aim of the study was to investigate the differences in the children’s performance between manual signs and PECS in terms of rates of acquisition, spontaneous use, retention, generalization, eye contact, and subsequent vocalization.

## 2. Materials and Methods

### 2.1. Participants

The data were collected by the therapist (during therapy sessions), by the teacher (at school) and by parents (at home). They shared the purposes and goals reached in the above-mentioned environments and recorded data on a single coding grid.

In this study, we considered 82 subjects diagnosed with ASD Level 1 and divided them into two groups of 41 subjects each. All the subjects had been recruited from the same city (Caserta, Italy) and were homogeneous in terms of the socio-cultural background of the parents; the family/environmental context did not represent an influencing factor on the level of education in either group. Therefore, the inclusion criteria were as follows: (a) aged between 20 and 24 months, (b) diagnosis of level 1 autism spectrum disorder in the absence of nosographically defined comorbidities, (c) absence of vocal language, and (d) medium-high socio-cultural class assessed through the SES scale [7].

The psychodiagnostic assessment was carried out by administering the ADI-R diagnostic interview to the parents [8], while the child was provided with a structured assessment according to the ADOS Toddlers module procedures [9]. After confirming the diagnosis and the possibility of inclusion in the sample, we divided the subjects into two experimental groups consisting of 41 subjects each. The subdivision was randomized. The subjects of both groups had the same inclusion criteria and did not have different sociocultural factors. The two groups were provided with the two different types of treatment, as will be discussed in the next paragraph. The first experimental group is composed of 41 subjects with an average age of 22.5 (M_age_ = 22.5, SD 0.21) and an average SES index of 7.22 (Ises 7.22, SD 1.03), of which 30 are males and 11 females. The second experimental group is composed of 41 subjects with an average age of 21.9 (M_age_ = 21.9, SD 0.39) and an average SES index of 7.18 (Ises 7.22, SD 1.19), of which 31 are males and 10 females (M_age_ = 14). Therefore, there were no differences in age or sociocultural level in the two groups. The data were collected at the FINDS Neuropsychiatry Outpatient Clinic by licensed psychologists in collaboration with the University of International Studies of Rome (UNINT).

In this study, we used two different AAC training methods in order to improve functional communication skills and speed up the acquisition of vocal language. The required AAC was used because it is the first communication phase of the young children analyzed. Children produce behaviors that have the value of signaling and requesting in order to achieve their goals (use of the vocal behavior in question).

### 2.2. Assessment Protocol

The protocol used for the psychodiagnostic assessment consists of the following tests: ADI-R (Autism Diagnostic Interview-Revised) [8], and ADOS 2—Toddler Module (Autism Diagnostic Observation Schedule) [9].

ADOS 2—Toddler Module: standardized semi-structured observation, aimed at evaluating communication and mutual interaction. It involves the administration of 11 activities to be presented to the child in the presence of a caregiver and lasts 30–45 min. The Toddler Module has been developed for children up to 30 months of age who are able to walk independently, with limited language and a chronological and non-verbal age of at least 12 months. The Toddler Module follows a structure similar to the other in that it must be administered in a room set up specifically for children and parents must always be present during the administration. Both cause and effect toys, as well as representational and imaginative toys, are included. The child’s ability to behave appropriately, given the needs of particular situations (fun shared with the adult, appropriate requests, seeking the other) is evaluated.

ADI-R (Autism Diagnostic Interview-Revised): semi-structured interview, addressed to caregivers, composed of 93 items, which investigates current and adopted behaviors between 4 and 5 years of age and helps to identify: anomalies in mutual social interaction, qualitative anomalies in communication, repetitive and stereotyped restricted behavior patterns. It focuses on the systematic and standardized observation of behaviors that are rarely found in non-clinical subjects, and mainly on the following three areas of functioning: language and communication, reciprocal social interaction, stereotyped behaviors and restricted interests. The ADI-R is divided into an interview protocol and five algorithms, which can be used at various ages for diagnosis or for surgery. If the purpose of the evaluation is to formulate a formal diagnosis, one of the two diagnostic algorithms is used (2–3–11 years; 4 years or more). If, on the other hand, the goal is to plan a therapy or educational project, one of the three algorithms of current behavior is used (3–11 years; 4–11 years; 10 years and over). The protocol used for the training consists of PECS and MS.

### 2.3. Procedures

The two training systems used are:

**Picture Exchange Communication System (PECS):** the PECS, developed in the United States in 1985 by Andy Bondy, Ph.D., and Lori Frost, is based on a PECS teaching protocol derived from Skinner’s text, *Verbal Behavior*; it provides a broad-spectrum application of the principles of applied behavioral analysis (ABA). It employs teaching and reinforcement strategies that intend to promote greater independence and systematic error correction procedures that can facilitate learning even in the event of an error. The PECS protocol consists of six phases and begins with teaching the student to deliver a single image to a “communication partner”, then goes on to teach the student how to discriminate images from each other and how to combine them to structure a sentence, while, in the more advanced stages, how to use attributes, answer questions and comment are introduced. The main focus of PECS is to teach functional communication; however, research shows that students who use PECS often also learn to speak [10]. The PECS training consists of six phases. Phase (1): The training begins on a single image of the highly desired object. Phase (2): A communication book is introduced, and a greater distance is placed between the child and the communication partner. Step (3): The child is required to discriminate between two image symbols (highly desired and unwanted elements to gradually obtain more desired elements). In Step (3), correspondence checks are carried out to ensure that the child truly requests the preferred item. Step (4): The child uses an opening sentence (“I want”) to make a request by constructing and exchanging a two-sentence stripe with a sequence of images with the symbol “I want” plus the image symbol for the favorite item. Step (5): The communication partner introduces the verbal prompt “What do you want?” Over time, a delay is inserted between the verbal request and an additional gesture request to the “I want” image symbol. Step (6): Comments are formed as children exchange phrases to answer their partner’s communicative questions (e.g., “What do you see?” “What do you want?” “What do you have?”) [11].

**Manual Signs (MS):** This was the first form of augmentative communication introduced for non-vocal people with developmental disabilities [12]. Firstly, although many people with developmental disabilities do not seem to have control over their vocal cords, they can often mimic actions. Secondly, modeling the signs allows you to use the physical prompt that promotes rapid learning without errors. Thirdly, the signs can be approximated and often adapted to the student’s level of praxis. Moreover, manual signs are easily generalizable to the child’s life context. Specifically, it is a combination of hand configurations that represent an expression, word, letter, number or combination of words. In both cases, two goals were identified: (a) achievement of 20 generalized spontaneous requests in three different life contexts (therapy, home and school), and (b) transfer to spontaneous vocal requests through generalized single words in three life contexts (therapy, home and school). Specifically, group 1 attended PECS training (Gr1/PECS) and was exposed to 15 h a week of ABA intervention with two additional coaching hours dedicated to parents for training on communication and domestic autonomy; for group 2, manual signs system training (Gr2/MS) was provided for 15 h a week using ABA intervention, as well as two additional coaching hours dedicated to parents for training on communication and domestic autonomy.

Before starting the training, all the children were independently assessed in naturalistic contexts. In particular, on skills related to the use of AAC/PECS, AAC/MS and the voice channel. The starting condition for both groups of subjects was a zero score on all three variables considered. Training: Each child underwent 15 h of ABA per week with 3 daily AAC sessions of 20 min each. In addition, the parents of the children of both groups were supported with 2 h a week of specific training focused on communication and autonomy (parent coaching). The first aim taken into consideration was the achievement of 20 spontaneous requests for generalized objects in three different contexts (therapy, home, school): in the case of the first group (Gr1 PECS), the goal was represented by the use of desired objects with 20 images, which in phase 3 were generalized to the three environmental contexts; in the case of the second group (Gr2 MS), the goal was represented by the use of desired objects of 20 spontaneous signs generalized to the three environmental contexts. The second goal was the transfer to vowel and the relative generalization for the 20 spontaneous requests acquired in AAC for both groups. For each goal, the parameter taken into consideration was the time taken to generalize the goal (Table 1).

Manual signs correspond to the images shown in PECS (Table 2). Moreover, manual signs were reported by the ISL (Italian Sign Language). If the child had praxis-motor difficulties, the manual sign was adapted to the child’s abilities.

## 3. Results

Data analyses were performed using SPSS 26.0 [13] statistical survey software. Significance was accepted at the 5% level (α < 0.05). We named Gr1 (group 1 who trained using PECS) and Gr2 (group 2 who trained using MS), Gen 1 (the goal of generalizing 20 spontaneous requests) and Gen 2 (the goal of generalizing 20 transfers to spontaneous vocal requests).

The comparison of the means of the groups was initially carried out using Student’s t-test, a parametric statistical test that can be used when the two groups in comparison are independent of each other. Specifically, we used the t-test for independent samples, in order to make comparisons between the two groups, with two-tailed significance.

In particular, we compared the times of Gen 1 between Gr1 and Gr2 and significant differences emerged in Gr1 [t = −54.318; *p* < 0.05]. These data show that the goal of generalizing 20 spontaneous requests in AAC is quicker in GR1 or when using the PECS system. We then compared the times of Gen 2 between Gr1 and Gr2 and significant differences emerged in Gr2 [t = 54.124; *p* < 0.05]. These data show that the goal of generalizing 20 transfers to spontaneous voice requests from AAC presents a shorter time in the GR2 or when using the system of signs (Table 3).

Subsequently, for a more in-depth analysis and to identify the dimension of the effects, we performed two ANOVAs at ONE VIA. In doing so, we were able to compare the average generalization times of the two goals (Gen 1 and Gen 2)—first in Gr1 and then in Gr2—and analyze any differences.

This analysis showed significant results in relation to Gen1 [F (1,81) = 2950,465, *p* < 0.05] in Gr1, confirming that the goal of generalizing 20 spontaneous requests in AAC presents a shorter time in GR1 or when using the PECS system. This analysis also highlighted significant results relative to Gen2 [F (1,81) = 2929,389, *p* < 0.05] in Gr2, confirming that the goal of generalizing 20 transfers to spontaneous voice requests from AAC presents a shorter time in Gr2 or when using the system of signs (Table 4).

## 4. Discussion

Recent studies investigating neural plasticity in the brains of very young children highlighted the importance of individualizing early treatment as a specific form of communication training [14]. Additionally, children with autism who learn to communicate exhibit lower levels of negative behaviors such as self-stimulation, self-harm and aggression [15]. Finally, there is no homogeneity in the scientific literature in comparisons of different types of AAC such as PECS and MS [10]. We compared these two methods of AAC (PECS and manual signs) with 82 children with autism aged 27 and 36 months and found that (a) children learned to use PECS faster than manual signs and (b) more children who used PECS were able to generalize this use to new elements than those who used manual signs. In our study, we investigated the correlation between these two forms of AAC. Specifically, we tried to find which of the two types of training provided the participants with a more immediate spontaneous use of some specific objects; moreover, the voice transfer and the relative generalization of the acquired spontaneous requests were improved. Our results revealed that the group that received PECS training showed a greater speed of acquisition/generalization of spontaneous requests; despite this, the participants undergoing MS training demonstrated a greater speed in acquiring the proposed tasks. It could be hypothesized that, in relation to PECS training, images may be easier for very young children to learn than manual signs for several reasons. First, images resemble their referents more than manual signs, making them easier to understand. Secondly, pointing or reaching at an image to make a request involves less physical effort and motor planning than performing a manual sign, and thirdly, images are made simple by reducing the memory load [16]. Subsequently, manual signs require participation (i.e., eye contact and attention) and imitation, both of which are skills that can be impaired and take a long time to acquire in autism [5]. These data influence the effectiveness of this type of treatment. Despite this, some authors [17,18] emphasize the importance of the gestural component of manual signs. Communication through gestures has been positively associated with the use of the voice in typical and autistic subjects and it is considered a precursor of language development [17,18]. Moreover, according to our findings, manual signs are strictly connected to imitation skills and imitation and language processing are closely connected. In addition, it is now established that Broca’s area, previously considered a speech production area, contains neurons activated by execution/observation/imitation [19,20].

These findings converge with the results we found, i.e., the goal of generalizing and transferring spontaneous voice requests takes less time in the group undergoing MS training.

Compared to other forms of AAC, signs are distinguished by their practicality since, while other methods require the use of devices or the retrieval of images or symbols, the hands are always available [19,20]. So, if the child shows interest in a new object, game or activity, the corresponding sign can be shown immediately. According to Tincani [21], choosing one AAC tool over another is not easy, and it is unlikely that a single system can best meet the needs of each autistic child and/or individuals with other disabilities. Furthermore, it is likely that a tool will be useful or not secondary to the child’s cognitive status. On the basis of the individual characteristics, it is, therefore, possible to utilize the most appropriate Augmentative Alternative Communication tool. Clearly, both systems in question have advantages and disadvantages. All AAC tools are characterized, for example, by their crucial role in breaking down communication barriers and consequently relational barriers, thanks to the overcoming of problem behaviors that usually emerge in these situations, such as frustration, despair, sense of loneliness and sometimes aggressive and oppositional attitudes. Tincani [21] demonstrates how the acquisition of manual signs is limited when the autistic child has imitative and motor difficulties. In the absence of these, it is more appropriate to use PECS. Manual signs are characterized by practicality and naturalness, distinguishing themselves considerably from the image exchange system [22]. Furthermore, compared to static images, on the other hand, manual signal can convey even complex concepts such as, for example, emotions. However, it has no scientific basis. Several studies have shown that proposing manual signs such as AAC does not harm oral production; on the contrary, they seem to favor the gradual increase in speech production in children and young people with communication disabilities with ASD [22,23]. It is possible to identify two different approaches. Some specialists argue the importance of introducing manual signs since the grammatical characteristics of manual signs seem to facilitate understanding [19]; on the other hand, there are those who consider it more appropriate to use AAC, as it requires the development of specific skills, which are often absent or reduced in children with communication difficulties. In any case, it is preferred to carefully analyze the individual characteristics of autistic subjects (cognitive, motor, imitative, discriminatory abilities). Manual signs, images, or a succession of symbols illustrating how to perform a task are easier to understand and interpret than words.

## 5. Conclusions

In conclusion, more studies comparing methods of AAC in children with autism (or other disabilities) are needed. The lack of studies that investigate this form of alternative communication and the comparison between its methods do not allow us to confirm specific conclusive results.

However, although PECS is more effective than manual signs in clinical practice, our study emphasizes the efficiency of both tools but in different processes. Future studies could take into account the idea that the variety of AAC methods can be effective when caregivers respond consistently and contingently to their children’s attempts at communication. In conclusion, more studies are suggested to favor the generalization of the results.

## Figures and Tables

**Table 1:** Description of training and procedures.

Group 1 (PECS)	Group 2 (MS)
Assessments of basic skills on AAC/MS and the voice channel in a natural context.	Assessments of basic skills on AAC/MS and the voice channel in a natural context.
15 h of ABA per week with 3 daily 20 min AAC sessions	15 h of ABA per week with 3 daily 20 min AAC sessions
Parent coaching: focused on communication and autonomy	Parent coaching: focused on communication and autonomy
Use of the desired objects of 20 images generalized to the three environmental contexts	Use for desired objects of 20 spontaneous signs generalized to the three environmental contexts
Transfer to the voice and the relative generalization for the 20 spontaneous requests acquired in AAC.	Transfer to the voice and the relative generalization for the 20 spontaneous requests acquired in AAC.

**Table 2:** PECS images.

Auditory	stereo, xylophone, piano, guitar, drums
Visual	soap bubbles, books, watercolors, cars, balls
Kinesthetic	jumping, tickle, modeling dough, slime, shaving cream
Edible	candy, chocolate, chips, cookies, lollypop

**Table 3:** Comparisons of the average generalization times by T TEST.

	Gr1		Gr2			
	Means	SD	Means	SD	t	*p*
Gen 1	22.56	1.20	38.92	1.50	−54.318	<0.05 *
Gen 2	51.90	1.47	37.97	0.72	54.124	<0.05 *

* Statistical significance *p* < 0.05.

**Table 4:** Comparisons of mean generalization times by ANOVA.

	Gr1		Gr2			
	Means	SD	Means	SD	F	*p*
Gen 1	22.56	1.20	38.92	1.50	2950.465	<0.05 *
Gen 2	51.90	1.47	37.97	0.72	2929.389	<0.05 *

* Statistical significance *p* < 0.05.

## Data Availability

The data presented in this study are available on request from the corresponding author.
